# Supplementary material for: Hybrid Models and Biological Model Reduction with PyDSTool
Source: PLoS Comput Biol. 2012 Aug 9;8(8):e1002628. doi: 10.1371/journal.pcbi.1002628 (PMC3415397; doi:10.1371/journal.pcbi.1002628)
Supplement: Text S4 — Complete source code for the PyDSTool package (version 0.88.120504). Includes API documentation and help files linking to web pages. This file is identical to the current public release on Sourceforge.net. (ZIP) [file pcbi.1002628.s004.zip › PyDSTool/html/toc-PyDSTool.Toolbox.synthetic_data-module.html]

xml version="1.0" encoding="ascii"?


synthetic\_data


# Module synthetic\_data

---

## Functions

generate\_ball

generate\_discspiral  
 generate\_hypercube  
 generate\_spiral  
 generate\_swirl  
 inf\_norm  
 max\_euclidean\_distance  
 maxs  
 mins  
 sup\_norm  

## Variables

ALLOW\_THREADS

API

API\_class  

Abs

Acos

Asin

Atan

Atan2

BUFSIZE

Betavariate

CLIP

Ceil

Choice

Continuous

Cos

Cosh

DO\_DEC  
 DO\_POW  

Degrees

Discrete

DomainType  

E

ERR\_CALL

ERR\_DEFAULT

ERR\_DEFAULT2

ERR\_IGNORE

ERR\_LOG

ERR\_PRINT

ERR\_RAISE

ERR\_WARN

E\_COMPUTFAIL

E\_NONUNIQUETERM

Exp

Expovariate

FLOATING\_POINT\_SUPPORT

FPE\_DIVIDEBYZERO

FPE\_INVALID

FPE\_OVERFLOW

FPE\_UNDERFLOW

Fabs

False\_

Floor

Fmod

Frexp

Gammavariate

Gauss

Getrandbits

Getstate

HAVE\_PSYCO

Hypot

Inf

Infinity

Jumpahead

LargestInt32

Ldexp

Log

Log10

Lognormvariate

MAXDIMS

MReg  
 Macheps  

Max

Min

Modf

NAMESEP  

NAN

NINF

NZERO

NaN

Normalvariate

ONES  

PINF

POW\_STR  

PZERO

Paretovariate

Pi

Pow

RAISE

Radians

Randint

Random

Randrange

SHIFT\_DIVIDEBYZERO

SHIFT\_INVALID

SHIFT\_OVERFLOW

SHIFT\_UNDERFLOW

Sample

ScalarType

Seed

Setstate

Shuffle

Sin

Sinh

Sqrt

Sum

Systemrandom

TENS  

Tan

Tanh

True\_

UFUNC\_BUFSIZE\_DEFAULT

UFUNC\_PYVALS\_NAME

Uniform

Vonmisesvariate

WRAP

W\_BISECTLIMIT

W\_NONTERMEVENT

W\_NONTERMSTATEBD

W\_TERMEVENT

W\_TERMSTATEBD

W\_UNCERTVAL

Weibullvariate

Wichmannhill

ZEROS  

\_generators

\_hypercuboid\_utils

absolute

accrueCompTypes  
 acosh  

add

allCompTypes  

allmathnames

allmathnames\_symbolic

alphabet\_chars\_RE  

arccos

arccosh

arcsin

arcsinh

arctan

arctan2

arctanh

asinh  
 ast2shortlist  
 ast2string  
 atanh  

bitwise\_and

bitwise\_not

bitwise\_or

bitwise\_xor

builtinFnSigInfo  
 builtin\_auxnames  
 builtinlookup  
 cPickle  

c\_

cast

ceil

collect\_numbers  
 complex  
 complex192  

conj

conjugate

contained

convert\_power\_reserved\_keywords  

copysign

cos

cosh

count\_sep  

deg2rad

degrees

divide

doadd  
 dodiv  
 dofun  
 domul  
 doneg  
 dopower  
 dosub  
 dsInterface  

e

ensurebare  
 ensuredecimalconst  
 ensureints  
 ensureparen  
 ensureparen\_div  

equal

errmessages

errorfields

exp

exp2

expm1

fabs

feature\_leaf  
 feature\_node  
 feval\_map\_const  
 feval\_map\_symb  
 findEndBrace  
 findNumTailPos  
 fit\_function  
 float96  

floor

floor\_divide

fmax

fmin

fmod

frexp

funcnames  

genDB

getdim  
 getlftrtD  

greater

greater\_equal

hypot

index\_exp

inf

infty

inverseMathNameMap

invert

isHierarchicalName  
 isIntegerToken  
 isNameToken  
 isNumericToken  
 isToken  
 isVectorClause  

isfinite

isinf

isnan

joinAsStrs  
 joinStrs  

ldexp

left\_shift

less

less\_equal

little\_endian

local\_fndef  

log

log10

log1p

logaddexp

logaddexp2

logical\_and

logical\_not

logical\_or

logical\_xor

makeParList  
 mapNames  
 mapPowStr  

mathNameMap

math\_dir  
 math\_globals  
 mathlookup  

maximum

mgrid

minimum

minpack  

mod

modf

modlookup  

multiply

n

nameResolver

name\_chars\_RE  

nan

nbytes

negative

newaxis

newton\_meth  

nextafter

not\_equal

notcontained

null\_predicate

null\_predicate\_class  
 num\_chars  

ogrid

ones\_like

parseMatrixStrToDictStr  
 parser  
 parserObject  

pi

power

predicate\_op  
 processMultiDef  
 process\_raw\_residual  
 proper\_match  

protected\_allnames

protected\_auxnamesDB

protected\_macronames  
 protected\_mathnames  
 protected\_randomnames  
 protected\_scipynames  
 protected\_specialfns  
 qtypes  

r\_

rad2deg

radians

randomlookup  
 re  
 readArgs  

reciprocal

regObject  

remainder

remove\_indices\_from\_range  
 replaceCallsWithDummies  
 replaceSep  
 replaceSepInv  
 replaceSepList  
 replaceSepListInv  
 replaceSepQSpec  
 replaceSepStr  

rerr

reserved\_keywords  
 resolveSpecTypeCombos  

right\_shift

rint

rout

s\_

sctypeDict

sctypeNA

sctypes

sign

signbit

simplify  
 simplify\_str  

sin

sinh

spacing

specTypes

specialfns  
 splitargs  
 splitastLR  

sqrt

square

strIfSeq  
 string2ast  

subtract

sym2name  
 symbol  
 symbolMapClass  

symbolMapDict

syms  

t

tan

tanh

targetLangs

temp\_macro\_names  
 temp\_macro\_names\_inv  

theGenSpecHelper

toCircumflexSyntax  
 toDoubleStarSyntax  
 toPowSyntax  
 token  
 traceback  

true\_divide

trunc

trysimple  
 typeCounter  

typeDict

typeNA

typecodes

uncertain

utils\_info  

verstr\_parts

warnfields

warnmessages

whoQ  

---

[hide private]
